# Death

**Published:** 1856-06

**Authors:** 


*Death.*

Dr. John E. Heald, of West Point, Indiana, died on the 29th of December, 1855, of Tubercular Phthisis. He was 46 years of age, and a man respected both as a practitioner and a citizen.

